# Lack of β_2_-adrenoceptors aggravates heart failure-induced skeletal muscle myopathy in mice

**DOI:** 10.1111/jcmm.12253

**Published:** 2014-03-13

**Authors:** Vanessa A Voltarelli, Luiz RG Bechara, Aline VN Bacurau, Katt C Mattos, Paulo MM Dourado, Carlos R Bueno, Dulce E Casarini, Carlos E Negrao, Patricia C Brum

**Affiliations:** a School of Physical Education and Sport, University of São PauloSão Paulo, Brazil; b Heart Institute, Faculty of Medicine, University of São PauloSão Paulo, Brazil; c School of Physical Education and Sport, University of São PauloRibeirão Preto, Brazil; d Department of Medicine, Division of Nephrology, Federal University of São PauloSão Paulo, Brazil

**Keywords:** heart failure, skeletal muscle, β_2_-adrenoceptors, proteasome

## Abstract

Skeletal myopathy is a hallmark of heart failure (HF) and has been associated with a poor prognosis. HF and other chronic degenerative diseases share a common feature of a stressed system: sympathetic hyperactivity. Although beneficial acutely, chronic sympathetic hyperactivity is one of the main triggers of skeletal myopathy in HF. Considering that β_2_-adrenoceptors mediate the activity of sympathetic nervous system in skeletal muscle, we presently evaluated the contribution of β_2_-adrenoceptors for the morphofunctional alterations in skeletal muscle and also for exercise intolerance induced by HF. Male WT and β_2_-adrenoceptor knockout mice on a FVB genetic background (β_2_KO) were submitted to myocardial infarction (MI) or SHAM surgery. Ninety days after MI both WT and β_2_KO mice presented to cardiac dysfunction and remodelling accompanied by significantly increased norepinephrine and epinephrine plasma levels, exercise intolerance, changes towards more glycolytic fibres and vascular rarefaction in plantaris muscle. However, β_2_KO MI mice displayed more pronounced exercise intolerance and skeletal myopathy when compared to WT MI mice. Skeletal muscle atrophy of infarcted β_2_KO mice was paralleled by reduced levels of phosphorylated Akt at Ser 473 while increased levels of proteins related with the ubiquitin-–proteasome system, and increased 26S proteasome activity. Taken together, our results suggest that lack of β_2_-adrenoceptors worsen and/or anticipate the skeletal myopathy observed in HF.

## Introduction

Heart failure (HF) is a complex syndrome involving multiple systems and neurohumoral compensatory mechanisms accompanied by high morbidity and mortality, and it is characterized by clinical signs, such as fatigue, dyspnoea and exercise intolerance [1,2]. Although HF is a syndrome of cardiac origin, it leads to skeletal muscle atrophy, a co-morbidity that is associated with poor prognosis [3–5].

Short-term increases in sympathetic activity lead to increases in skeletal muscle mass. Conversely, chronic sympathetic hyperactivity is detrimental, contributing to muscle wasting in HF, as demonstrated previously [6]. In fact, sympathetic hyperactivity in HF could decrease the density and sensitivity of skeletal muscle β_2_-adrenoceptor (β_2_-AR), reducing anabolic and anti-catabolic stimuli promoted by these receptors on skeletal muscle fibres.

It is known that β_2_-AR subtype is highly prevalent (∽90%) in skeletal muscle and plays a key role in regulating skeletal muscle mass in both anabolic and catabolic conditions [7]. The activation of these receptors leads to increased protein synthesis and inhibition of protein degradation pathways [8,9]. Furthermore, we have shown recently that β_2_-ARs are also involved in endurance capacity [10]. However, the involvement of β_2_-AR signalling in skeletal muscle morphology and functional capacity changes observed in HF is not known yet. Taking into consideration that β_2_-ARs mediate the activity of sympathetic nervous system in skeletal muscle and that sympathetic hyperactivity is one of the main components involved in HF-induced skeletal myopathy [11], one would suggest that chronic activation of β_2_-AR is associated with skeletal muscle morphofunctional alterations in HF.

In the present study, we tested the hypothesis that lack of β_2_-ARs would aggravate skeletal myopathy and exercise intolerance in myocardial infarction (MI)-induced HF mice. For doing that, we have submitted β_2_-adrenoceptors knockout mice (β_2_KO) in FVB genetic background to MI or SHAM surgery to evaluate the contribution of β_2_-ARs on morphofunctional alterations in skeletal muscle and also on exercise intolerance induced by HF. The key findings of the present study are that MI-induced cardiac dysfunction and remodelling in both WT and β_2_KO mice, which was accompanied by significantly increased norepinephrine and epinephrine plasma levels, exercise intolerance, changes in muscle fibre type and vascular rarefaction in plantaris muscles. However, infarcted β_2_KO mice displayed a higher decrease in exercise tolerance and more severe skeletal myopathy associated with reduced levels of phosphorylated Akt at ser473, increased levels of proteins related with the ubiquitin–proteasome system (UPS), and increased 26S proteasome activity.

## Materials and methods

### Animal model and experimental design

Four-month-old male FVB mouse strain lacking β_2_-adrenoceptor (β_2_KO) [12] and their age-matched wild-type controls (WT) were randomly assigned into MI or fictitious surgery (SHAM). Mice were deeply anaesthetized with ketamine (80 mg/kg, *ip*) and xylazine (6 mg/kg, *ip*), followed by left thoracotomy and ligation of the left anterior descending (LAD) coronary artery. SHAM operated animals underwent the same surgical procedures, except for LAD ligation. Three months after surgical procedures, mice were submitted to echocardiographical assessment, exercise testing and ambulation test for assessment of functional capacity, as described below.

Wild-type SHAM group refers to FVB mice, fictitiously operated control group, WT MI group refers to three-month post-MI FVB mice, β_2_KO SHAM group refers to fictitiously operated β_2_KO mice, and β_2_KO MI group refers to three-month post-MI β_2_KO mice. The animals were maintained at School of Physical Education and Sport of University of São Paulo, in a 12:12-h dark–light cycle and a temperature-controlled environment (22°C) with free access to standard laboratory chow (Nuvital Nutrients, Curitiba, Brazil) and tap water. All procedures were performed in accordance with the Guide for the Care and Use of Laboratory Animals (National Institutes of Health, Bethesda, MD, USA) and with ethical principles in animal research adopted by the Brazilian College of Animal Experimentation (www.cobea.org.br). In addition, this study was approved by University of São Paulo-EEFE Ethical Committee (CEP no. 2008/53).

### Echocardiographical evaluation

Non-invasive cardiac function was assessed by two-dimensional guided M-mode echocardiography, in ketamine- (80 mg/kg) and xylazine (6 mg/kg)-anaesthetized WT and β_2_KO mice. Briefly, mice were positioned in the supine position and an ultrasound transmission gel was applied to the precordium. Transthoracic echocardiography was performed using Sequoia 512 echocardiography machine (Acuson, Mountain View, CA, USA) equipped with a 14-MHz linear transducer. Heart rate was kept similar in all groups studied during the evaluation to avoid artifactual changes in left ventricle fractional shortening (FS). Left ventricle systolic function was estimated by FS as follows: FS (%) = [(LVEDD − LVESD)/LVEDD] × 100, where, LVEDD means left ventricular end-diastolic diameter, and LVESD means left ventricular end-systolic diameter.

### Graded treadmill exercise test

Exercise capacity, estimated by total distance run, correlates with skeletal muscle work capacity. Exercise capacity was evaluated using a graded treadmill exercise protocol for mice as previously described [13]. Briefly, after being adapted to treadmill exercises over a week (10 min. of exercise per session), mice were placed in the treadmill lane and allowed to acclimatize for at least 30 min. Intensity of exercise was increased by 3 m/min. (6–45 m/min.) every 3 min. at 0% grade, with no electric shock, until exhaustion. Exhaustion was defined as the moment when animals were unable to keep in pace with the treadmill for up to 1 min.

### Skeletal muscle functional assessment

Mice were submitted to the ambulation test, which determined the mean step length, measured in hind foot ink prints while mice ran freely in a corridor (length 50 cm; width 8 cm; height of lateral walls 20 cm) [14]. The mice were subjected to three successive trials, and the performance of each animal was measured as its best individual performance over the three trials [15].

### Catecholamine measurements

Plasma norepinephrine and epinephrine were measured by HPLC using ion-pair reverse-phase chromatography coupled with electrochemical detection (0.5 V), as previously described [16].

### Plantaris fibre typing and cross-sectional area

Forty-eight hours after the graded treadmill exercise test, mice were killed and plantaris muscles were harvested, immediately frozen in melting isopentane and stored in liquid nitrogen. Frozen muscles were cut into 10-μm cross-sections from the proximal to distal region using a cryostat (Micron HM505E; Zeiss, Walldorf, Germany). Muscle sections were then incubated for myofribrillar ATPase activity after alkali (myosin ATPase, pH 10.3) or acid (myosin ATPase, pH 4.6) pre-incubation as previously described [17]. The myosin ATPase reaction was used to identify the muscle fibre type. Type I fibres reacted deeply after acid pre-incubation at pH 4.6 and lightly after formaldehyde pre-treatment and alkali pre-incubation at pH 10.3. The inverse occurred with type II muscle fibres. Fibre typing and fibre cross-sectional area (CSA) were evaluated in whole plantaris muscle (∽500 and 300 fibres) at ×200 magnification and further analysed on a digitizing unit connected to a computer (Image-Pro Plus; Media Cybernetics, Silver Spring, MD, USA). The total number of each fibre type was counted to calculate the numerical fibre type composition (I, IIA and IIB). The CSA of each fibre type was measured for further calculation of average fibre CSA. All analyses were conducted by a single observer (V.A.V.) blinded to mouse's identity.

### Capillary-to-fibre ratio

Capillary-to-fibre ratio of plantaris muscle was evaluated after myofibrillar ATPase histochemistry reaction at pH 10.3 as previously described [18]. Briefly, capillary-to-fibre ratio was quantified by a 10 × 10 grid optically superimposed on each of five non-overlapping fields at ×400 magnification, distributed in a random manner using a computer-assisted morphometric system (Quantimet 500; Leica, Cambridge, UK). For calculating capillary-to-fibre ratio, the total number of capillaries was divided by the total number of fibres counted in the same field for whole plantaris (∽500 fibres). Only vessels with a diameter <10 μm were counted, which would largely comprise capillaries but might also include terminal arterioles or venules. All analyses were conducted by a single observer (V.A.V.) blinded to mouse's identity.

### Skeletal muscle protein expression

Immunoblots of WT SHAM, WT MI, β_2_KO SHAM and β_2_KO MI plantaris muscle homogenates were performed as described [19]. Briefly, liquid nitrogen-frozen muscles were homogenized in a buffer containing Mannitol 210 mM, Sucrose 70 mM, MOPS 5 mM, EDTA 1 mM, 0.1%Triton ×100, pH 7.4 and protease inhibitor cocktail (1:100; Sigma-Aldrich, St. Louis, MO, USA). Samples were loaded and subjected to SDS-PAGE (8%, 10% and 12% gel percentages, depending on the protein). After electrophoresis, proteins were electrotransferred to nitrocellulose membrane (Amersham Biosciences, Piscataway, NJ, USA). Equal loading of samples (25 μg) and even transfer efficiency were monitored with the use of 0.5% Ponceau S staining of the blotted membrane. The blotted membrane was then incubated in a blocking buffer (5% bovine serum albumin, 10 mM Tris·HCl, pH 7.6, 150 mM NaCl and 0.1% Tween 20) for 2 hrs at room temperature and then incubated with a specific antibody overnight at 4°C. Antibodies to Protein Kinase B phosphorylated at ser473 (p-Akt ser473; Cell Signaling Technology, Beverly, MA, USA), Protein Kinase B (Akt1; abcam®, Cambridge, UK), Eukaryotic translation initiation factor 4E-binding protein 1 (4E-BP1; Cell Signaling Technology), Eukaryotic translation initiation factor 4E-binding protein 1 phosphorylated at thr70 (p-4E-BP1 thr70; Cell Signaling Technology), Eukaryotic translation initiation factor 4E-binding protein 1 phosphorylated at thr37/46 (p-4E-BP1 thr37/46; Cell Signaling Technology), Forkhead Box O3 (FoxO3a; Cell Signaling Technology), Forkhead Box O3 phosphorylated at ser253 (p-FoxO3a ser253; Cell Signaling Technology), F-box protein 32 (Atrogin-1, Anti-Fbx-32; abcam®), Muscle RING-finger protein-1 (MuRF-1; Santa Cruz Biotechnology, Dallas, TX, USA), Protein ubiquitination (Ubiquitin Antibody; Santa Cruz Biotechnology, Santa Cruz, CA, USA). Binding of the primary antibody was detected with the use of peroxidase-conjugated secondary antibodies (anti-rabbit IgG e antimouse IgG; Cell Signaling Technology, for 1.5 hr at room temperature) and detection was performed in a digitalizing unit (ChemiDoc; BioRad, Hercules, CA, USA) after incubation with luminol and hydrogen peroxide as HRP substrate. Quantification analysis of blots was performed using ImageJ software (U.S. National Institutes of Health, Bethesda, MD, USA). Targeted bands were normalized to Glyceraldehyde 3-phosphate dehydrogenase (GAPDH; Cell Signaling Technology). Skeletal muscle protein ubiquitination was analysed in the broadest molecular weight range as possible and results were corrected to Ponceau red staining (0.5%, w:v) of the membrane. Data are presented as percentage of SHAM group (arbitrarily set as 100%). Representative blots are provided along with the data.

### Proteasomal activity

Muscles were homogenized in a buffer containing Mannitol 210 mM, Sucrose 70 mM, MOPS 5 mM, EDTA 1 mM, 0.1% Triton ×100, pH 7.4, in absence of protease inhibitors and centrifuged for 15 min. at 12,000 × g and 4°C, pellet was discarded and supernatant (soluble proteins) was used for the assay. 26S Proteasome activity was measured as the cleavage rate of a synthetic fluorescent peptide (Suc-LLVY-AMC, product #P802-0005 from Enzo Life Sciences, Farmingdale, NY, USA), a specific substrate of proteasome chymotrypsin catalytic site. Reaction mixture contained 25 mM Tris (pH 7.4), 5 mM MgCl_2_, 25 μM ATP, 25 μM LLVY-AMC and the sample (25 μg of soluble proteins). Fluorescent product formation was followed up for 90 min. (440 and 350 nm were emission and excitation wavelengths respectively) at 37°C in the presence or absence of epoxomycin (20 μM), a highly specific inhibitor of chymotrypsin-like proteasome activity, and the difference between the two rates was considered as 26S proteasomal activity (chymotrypsin-site).

### Statistical analysis

All variables showed normal distribution, when analysed using the Shapiro–Wilk normality test, and therefore, the parametric statistical analysis was used. Data were expressed as mean ± SEM. Two-way anova with *post hoc* testing by Duncan (Statsoft Statistica software version 8.0; StatSoft, Inc., Tulsa, OK, USA) was used to compare the effect of genotype (WT and β_2_KO mice) and surgery (SHAM and MI). Statistical significance was considered achieved when *P* < 0.05.

## Results

### Physiological parameters

Physiological parameters are presented in Table1. β_2_-AR disruption had no impact on either bodyweight or plasma norepinephrine and epinephrine. However, β_2_KO SHAM displayed reduced heart-to-bodyweight ratio. MI, regardless of genotype, increased heart-to-bodyweight ratio paralleled by increased plasma norepinephrine and epinephrine levels. MI extension was similar between WT and β_2_KO mice.

**Table 1:** Physiological parameters

	WT		β_2_KO	
	SHAM (*n* = 14)	MI (*n* = 11)	SHAM (*n* = 14)	MI (*n* = 12)
Bodyweight (g)	33 ± 1	32 ± 2	33 ± 1	31 ± 1
HW/BW (mg/g)	3.8 ± 0.1	5.8 ± 0.3*	3.0 ± 0.1*,***	5.2 ± 0.4*,**
LW/BW (mg/g)	5.6 ± 0.1	7.1 ± 0.8*	5.7 ± 0.3	7.8 ± 0.6*,**
Plasma norepinephrine (ng/ml)	7.5 ± 0.7	12.6 ± 1.3*	8.0 ± 0.5***	10.9 ± 1.3*,**
Plasma epinephrine (ng/ml)	4.6 ± 1.2	16.6 ± 4.1*	6.9 ± 1.0***	13.1 ± 2.9*,**
Infarcted area (%)	–	35 ± 3	–	39 ± 4

Bodyweight, heart weight to bodyweight ratio (HW/BW), lung weight to bodyweight ratio (LW/BW), plasma norepinephrine and epinephrine content, and infarcted area of WT and β_2_KO mice submitted to fictitious surgery (SHAM) and myocardial infarction (MI).

Data are presented as mean ± SEM.

* *P* < 0.05 *versus* WT SHAM;

** *P* < 0.05 *versus* β_2_KO SHAM;

*** *P* < 0.05 *versus* WT MI.

β_2_-AR disruption did not alter left ventricle dimensions and contractility while MI induced severe contractile dysfunction (reduced LVFS) regardless of genotype (Table2). Of interest, MI induced LV dilation, at both systole and diastole, which was aggravated by β_2_-AR disruption. These data suggest that cardiac remodelling occurs along with contractile dysfunction in MI groups.

**Table 2:** Echocardiographical data

	WT		β_2_KO	
	SHAM (*n* = 14)	MI (*n* = 11)	SHAM (*n* = 14)	MI (*n* = 12)
LVFS (%)	48 ± 2	26 ± 2*	45 ± 1***	22 ± 1*,**
LVESD (mm)	1.65 ± 0.08	3.09 ± 0.13*	1.74 ± 0.06***	3.72 ± 0.10*,**,***
LVEDD (mm)	3.20 ± 0.12	4.18 ± 0.15*	3.15 ± 0.08***	4.78 ± 0.12*,**,***

Left ventricular fractional shortening (LVFS), left ventricular end-systolic diameter (LVESD) and left ventricular end-diastolic diameter (LVEDD) of WT and β_2_KO mice submitted to fictitious surgery (SHAM) and myocardial infarction (MI).

Data are presented as mean ± SEM.

* *P* < 0.05 *versus* WT SHAM;

** *P* < 0.05 *versus* β_2_KO SHAM;

*** *P* < 0.05 *versus* WT MI.

### Myocardial infarction induced plantaris myopathy and β_2_-AR disruption aggravates its functional capacity

**Figure 1:**
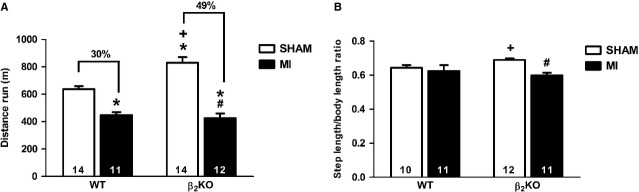
Total distance run in graded treadmill running test (A) and step length in ambulation test (B) of WT and β_2_KO mice submitted to fictitious surgery (SHAM) and myocardial infarction (MI). Data are presented as mean ± SEM. *P < 0.05 *versus* WT SHAM; #*P* < 0.05 *versus* β_2_KO SHAM; +*P* < 0.05 *versus* WT MI.

Functional capacity was assessed as maximal exercise performance in running test until exhaustion and ambulation test. Figure1 shows that β_2_KO SHAM mice displayed higher exercise tolerance than WT SHAM mice (Fig.1A) with no difference in step-to-body length ratio (Fig.1B). Myocardial infarction induced exercise intolerance in both β_2_KO MI and WT MI mice when compared with their respective SHAM control mice; however, the magnitude of distance run reduction was greater in β_2_KO than WT MI mice (49 ± 4 *versus* 30 ± 3%, *P* < 0.05; Fig.1A). Interestingly, only β_2_KO MI mice displayed decreased performance on ambulation test (Fig.1B), which suggests that β_2_-AR disruption aggravated MI-induced muscle strength loss.

### Fibre type distribution, capillary-to-fibre ratio and cross-sectional area

**Figure 2:**
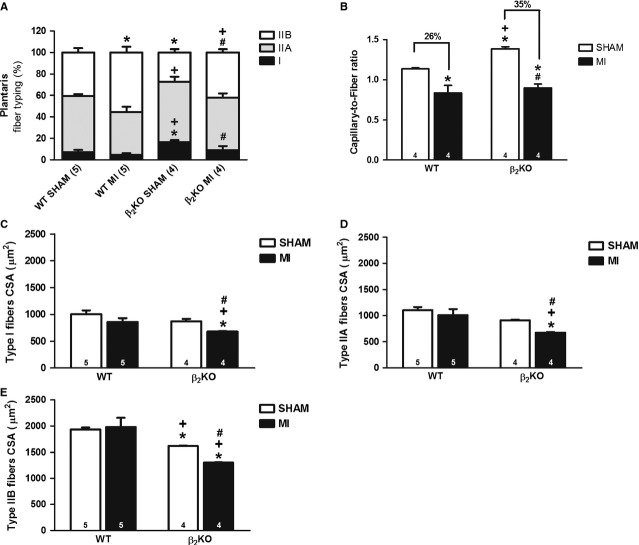
Plantaris muscle fibre type distribution (A) capillary-to-fibre ratio (B) and cross-sectional area (CSA) of types I, IIA and IIB fibres (C–E respectively) in WT and β_2_KO mice submitted to fictitious surgery (SHAM) and myocardial infarction (MI). Data are presented as mean ± SEM. *P < 0.05 *versus* WT SHAM; #*P* < 0.05 *versus* β_2_KO SHAM; +*P* < 0.05 *versus* WT MI.

In line with the improved exercise tolerance, β_2_-AR disruption induced a shift towards type I fibre in plantaris muscle paralleled by an increased capillary-to-fibre ratio and reduced plantaris type IIB fibre CSA (Fig.2A–E).

Myocardial infarction induced a shift towards more glycolytic fibres (Fig.2A) and capillary rarefaction (Fig.2B), however these responses were more pronounced in β_2_KO MI mice. Of interest, reduced plantaris type I, type IIA and type IIB CSA was restricted to β_2_KO MI mice, suggesting that lack of β_2_-ARs aggravated MI-induced skeletal muscle myopathy (Fig.2C–E).

### Skeletal muscle expression of proteins involved in Akt signalling pathway

**Figure 3:**
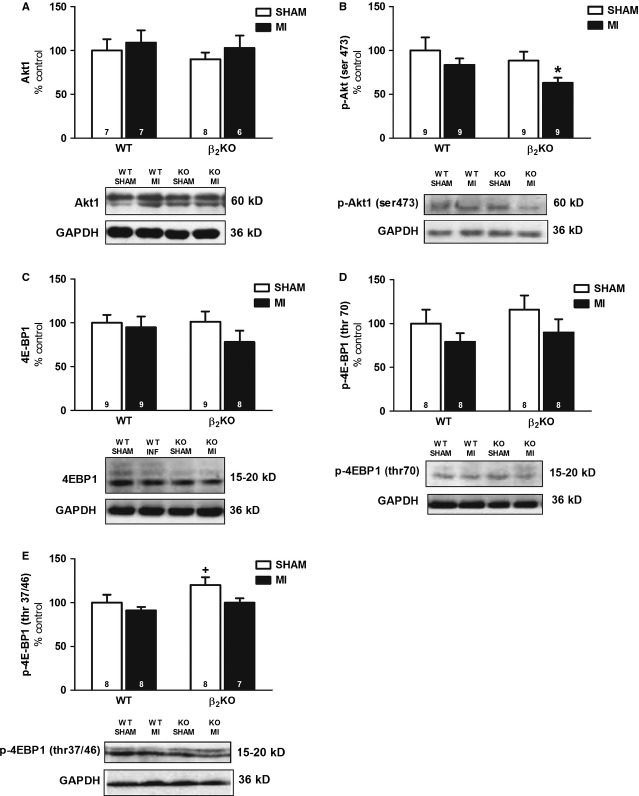
Plantaris expression of Protein Kinase B (Akt1, A), Protein Kinase B phosphorylated at ser473 (p-Akt ser473, B), Eukaryotic translation initiation factor 4E-binding protein 1 (4E-BP1, C), Eukaryotic translation initiation factor 4E-binding protein 1 phosphorylated at thr70 (p-4E-BP1 thr70, D), Eukaryotic translation initiation factor 4E-binding protein 1 phosphorylated at thr37/46 (p-4E-BP1 thr37/46, E) in WT and β_2_KO mice submitted to fictitious surgery (SHAM) and myocardial infarction (MI). Data are presented as mean ± SEM after normalization against Glyceraldehyde 3-phosphate dehydrogenase (GAPDH). *P < 0.05 *versus* WT SHAM; #*P* < 0.05 *versus* β_2_KO SHAM; +*P* < 0.05 *versus* WT MI.

Figure3 shows that the expression of proteins involved in Akt signalling was not affected by genotype, which suggests that the reduction in type IIB fibres CSA in β_2_KO SHAM mice was not related to Akt signalling pathway impairment. In contrast, MI decreased p-Akt protein levels (Fig.3B), which was restricted to β_2_KO MI mice when compared to WT SHAM mice. No changes were observed among groups in total 4EBP1, p-4EBP1 at thr70 while an increased p-4EBP1 at thr37/46 was observed in β_2_KO SHAM mice compared to WT MI.

### Skeletal muscle ubiquitin–proteasome system

**Figure 4:**
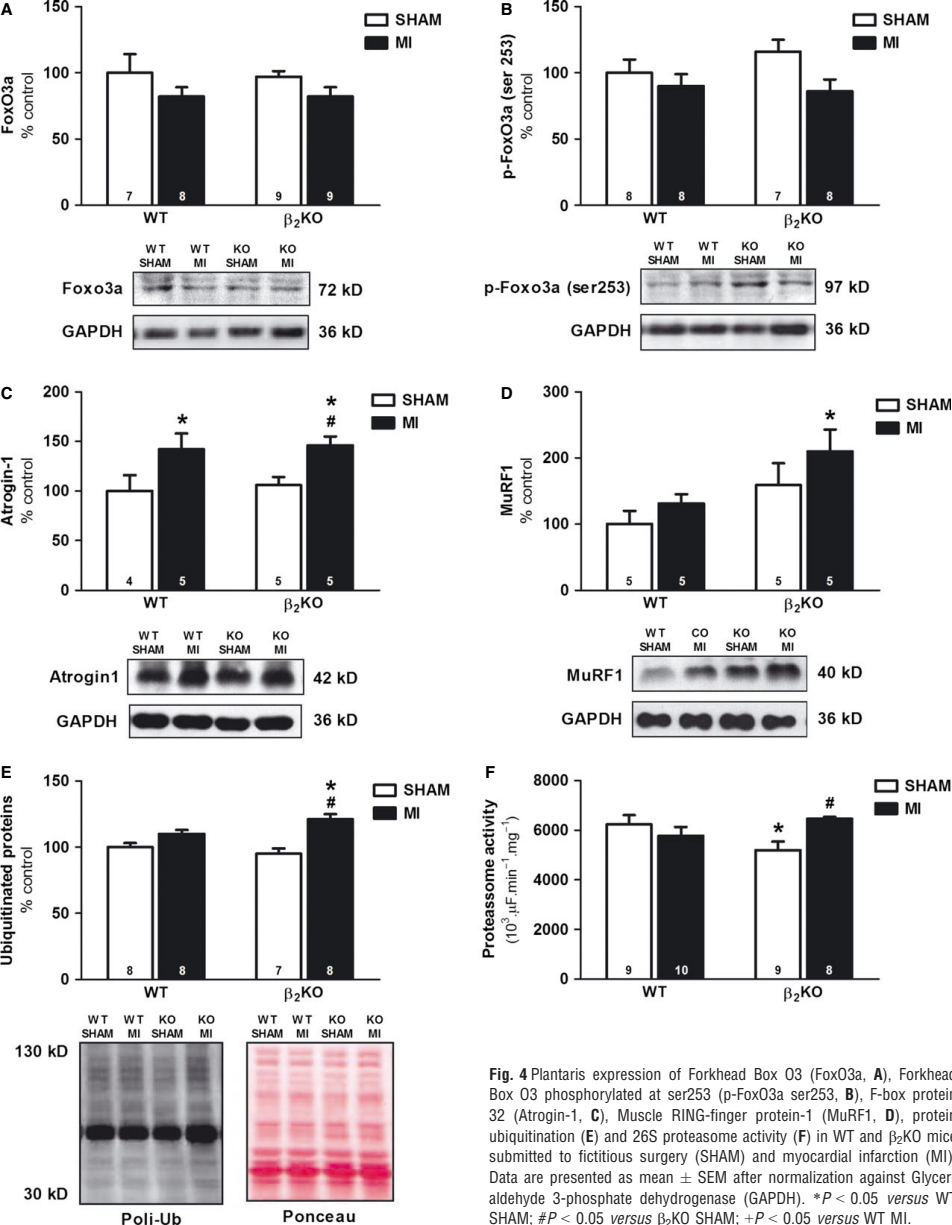
Plantaris expression of Forkhead Box O3 (FoxO3a, A), Forkhead Box O3 phosphorylated at ser253 (p-FoxO3a ser253, B), F-box protein 32 (Atrogin-1, C), Muscle RING-finger protein-1 (MuRF1, D), protein ubiquitination (E) and 26S proteasome activity (F) in WT and β_2_KO mice submitted to fictitious surgery (SHAM) and myocardial infarction (MI). Data are presented as mean ± SEM after normalization against Glyceraldehyde 3-phosphate dehydrogenase (GAPDH). *P < 0.05 *versus* WT SHAM; #*P* < 0.05 *versus* β_2_KO SHAM; +*P* < 0.05 *versus* WT MI.

Lack of β_2_-ARs had no impact on FoxO3a, p-FoxO3a, Atrogin-1, MuRF1 protein expression and protein ubiquitination (Fig.4A–E), whereas it decreased chymotrypsin-like proteasome activity (Fig.4F).

Myocardial infarction increased Atrogin-1 protein expression in both WT and β_2_KO mice (Fig.4C). Additionally, a significant increased MuRF1 expression was observed in β_2_KO MI compared with WT SHAM (Fig.4D). Figure4E shows that MI increased protein ubiquitination, a master signal for proteasomal degradation, was restricted to β_2_KO MI mice and accompanied by an increased chymotrypsin-like proteasome activity (Fig.4F).

## Discussion

Here, we report for the first time that lack of β_2_-ARs aggravates HF-induced skeletal myopathy in infarcted mice. The main findings of the present study are that MI associated with β_2_-AR gene targeting disruption induces: (*i*) more pronounced exercise intolerance when compared with WT MI group, (*ii*) skeletal muscle atrophy paralleled by decreased p-Akt protein levels and increased expression of proteins involved in UPS.

Exercise tolerance is a clinical predictor of HF syndrome and it is closely related to cardiac and skeletal muscle function [3,20]. Presently, a greater reduction in functional capacity was observed in β_2_KO MI than WT MI mice when both running until exhaustion and ambulation tests were assessed. β_2_-AR gene inactivation had no additional impact on MI-induced impaired left ventricle contractility while it aggravated plantaris myopathy. Even though the greater reduction in functional capacity of β_2_KO MI mice seems to rely on skeletal muscle abnormalities in the absence of β_2_-ARs, we cannot exclude that a minor reduction in cardiac output could also affect exercise tolerance. However, it is important to highlight that symptoms that typify HF, such as degree of exercise intolerance, are not directly related to the degree of cardiac dysfunction [3–5] but directly related to abnormalities in skeletal muscle and we presently extended this knowledge to the role of β_2_-AR signalling in this process. Indeed, we cannot exclude the possibility that part of this greater decrease in exercise tolerance of β_2_KO MI mice could be related to the reversal of the skeletal muscle adaptations underlying the higher baseline exercise tolerance observed in β_2_KO SHAM mice (*e.g*. greater soleus and plantaris percentage of oxidative fibres, capillarization, glycogen content and activity of oxidative enzymes) [10].

Chronic sympathetic hyperactivity is detrimental and might reduce β_2_-AR signalling in skeletal muscle contributing to installation of skeletal myopathy. In fact, we have observed reduction in β_2_-AR protein levels in WT MI when compared with WT SHAM mice (Figure S1) associated with increased norepinephrine and epinephrine plasma levels (Table1), which suggests that a reduced β_2_-AR signalling might precede the installation of HF-induced myopathy. This is of particular importance as β_2_-ARs are highly expressed (a ∽10-fold greater proportion than either β_1_ or β_3_ ARs) in skeletal muscle and regulate muscle contraction, metabolism and mass [21]. In line with these findings, β_2_-AR gene inactivation aggravated MI-induced plantaris myopathy in β_2_KO MI characterized by a more pronounced reduction in type I fibres and capillarity, and mainly skeletal muscle atrophy when compared with WT MI mice.

As β_2_-AR activation leads to skeletal muscle hypertrophy [7,8], its absence in a catabolic condition, such as HF, might have contributed for the induction of skeletal muscle atrophy observed in β_2_KO MI mice. In fact, the anabolic and anti-catabolic effects of β_2_-AR activation have been explained by its impact on the balance between protein synthesis and degradation. β_2_-AR by coupling to Gαi subunit and/or by activating cAMP/Epac pathway can further activate downstream pathways, such as Akt signalling that, when phosphorylated, increases protein synthesis while reduces protein degradation by UPS in skeletal muscle [8,22–24]. Accordingly, we presently observed that β_2_-AR disruption and MI were paralleled by a reduction in phosphorylated Akt protein levels (p-Akt ser 473, Fig.3B) and activation of UPS (Fig.4C–F) in plantaris muscle of β_2_KO MI mice. Thus, reduced p-Akt might be a mechanistic link between reduced β_2_-AR signalling and skeletal muscle atrophy in HF.

Akt activation leads to an increased protein synthesis through mTOR signalling or reduction in protein degradation through FoxO inhibition, which decreases atrogenes transcription, such as atrogin-1 and MuRF1 [25,26]. As we have observed no difference in 4EBP1 and p-4EBP1 protein levels, the skeletal muscle atrophy in β_2_KO MI mice does not seem to be modulated by an impaired protein synthesis. In contrast, decreased p-Akt protein levels observed in β_2_KO MI mice were paralleled by increased protein levels of atrogin-1, MuRF1 and ubiquitinated proteins, as well as by increased chymotrypsin-like proteasome activity. These data support an association between decreased p-Akt protein levels and the increased UPS activation in β_2_KO MI mice that could be mediated by FoxO transcription factors, as FoxO phosphorylation by Akt inhibits FoxO induction of atrogin-1 and MuRF1 transcription [25,26]. Even though we have not observed a significant decrease in phosphorylated FoxO3a protein levels (main isoform involved in skeletal muscle atrophy) in plantaris of β_2_KO MI mice, we cannot exclude that other FoxO isoforms could be involved in these responses. In fact, both FoxO1 and FoxO4 have also been implicated in the regulation of atrogin-1 and MuRF1 in catabolic processes [27–29]. Moreover, we cannot exclude that the greater decrease in phosphorylated FoxO3a in β_2_KO MI mice might have occurred prior to the stage of HF presently studied (90 days after MI).

The crucial role of UPS in HF-induced skeletal muscle atrophy observed in the present study corroborates our previous findings [30,31] and others [32–34]. In fact, UPS is considered the main proteolytic system involved in skeletal muscle atrophy [35,36], as it is responsible for cleaving about 90% of cytosolic proteins [37].

Altogether, our results give support to the notion that β_2_-AR disruption accelerates the installation of skeletal muscle atrophy in MI-induced HF. Considering that skeletal muscle atrophy and cachexia are related to poor prognosis and mortality in HF [38], it is relevant to drive more attention to skeletal myopathy in HF pharmacological therapy, particularly to β-blockers therapy, which is normally directed at improving cardiac function and do not counteract exercise intolerance or skeletal myopathy [6,39–41]. Even though the use of non-selective β-blockers with antioxidant and vasodilatory effects have impact on counteracting some features of HF-induced skeletal myopathy [42], the use of β_1_-AR selective β-blockers (cardiac action with minimal effect on skeletal muscle) with vasodilatory and antioxidant properties could have even higher effect counteracting skeletal myopathy considering that preserved β_2_-AR signalling could potentiate these peripheral effects. In fact, nebivolol has been shown to be effective with greater benefits than prior generations of β-blockers [43–45]. However, further studies are required to better understand the effect of different β-blockers normally used for HF therapy on skeletal myopathy.

## Study limitations

One might argue that the aggravated skeletal myopathy in β_2_KO mice could be related to a greater decrease in cardiac output in the absence of cardiac β_2_-ARs. Even though β_2_-AR gene inactivation had no impact on MI-induced impaired left ventricle FS and increased plasma norepinephrine and epinephrine levels, we cannot exclude that a minor decrease in cardiac output associated with greater left ventricle dimensions might have contributed to skeletal myopathy observed in β_2_KO mice.

Even though β_2_-AR is the predominant β-AR isoform expressed in skeletal muscle, one might suggest that, in the absence of β_2_-AR protein expression, up-regulation of β_1_-ARs could affect our results. This seems not be the case, since β_2_-AR gene inactivation did not alter plantaris β_1_-AR protein levels in both SHAM and MI groups (Figure S2).

In the present study, we focused on the skeletal muscle β_2_-AR signalling controlling skeletal muscle mass to better understand its role in HF-induced skeletal myopathy. However, β_2_-ARs are also expressed in vessels and its activation induces smooth muscle relaxation and endothelium-derived nitric oxide release [46,47]. However, β_1_-AR is the main β-AR subtype involved in vasodilation of resistance vessels (femoral artery and vein) [48] while both β_1_- and β_2_-ARs [48,49] are involved in mediating vasodilation in conductance arteries (thoracic aorta). Of interest, we previously reported similar β_1_-AR immunostaining in aorta of WT and β_2_KO mice [49]. Therefore, if β_2_-AR gene inactivation leads to changes in vessel smooth muscle tone, these changes would be negligible.

## Conclusion and perspective

Taken together, we provide evidence that inactivation of β_2_-AR aggravates MI-induced plantaris myopathy and anticipates plantaris atrophy associated with increased UPS activation. Altogether, we provide new insights on the molecular mechanisms whereby β_2_-AR can counteract skeletal myopathy in HF, which should be taken into consideration for the use of selective *versus* non-selective β-AR subtype antagonist therapy in HF.

## Supporting Information

**Figure S1** Plantaris β_2_-AR protein levels in WT mice submitted to SHAM or MI surgery. Data are presented as mean ± SEM. **P* < 0.05 *versus* WT SHAM (Unpaired Student's *t*-test).

**Figure S2** β_1_-AR protein levels in plantaris muscles of WT and β_2_KO mice from SHAM and MI groups. Data are presented as mean ± SEM.

**Data S1** Plantaris protein expression of β_2_-AR and β_1_-AR.
