# Supplementary figures and images for: Lack of β2-adrenoceptors aggravates heart failure-induced skeletal muscle myopathy in mice
Source: J Cell Mol Med. 2014 Mar 13;18(6):1087–97. doi: 10.1111/jcmm.12253 (PMC4508148; doi:10.1111/jcmm.12253)

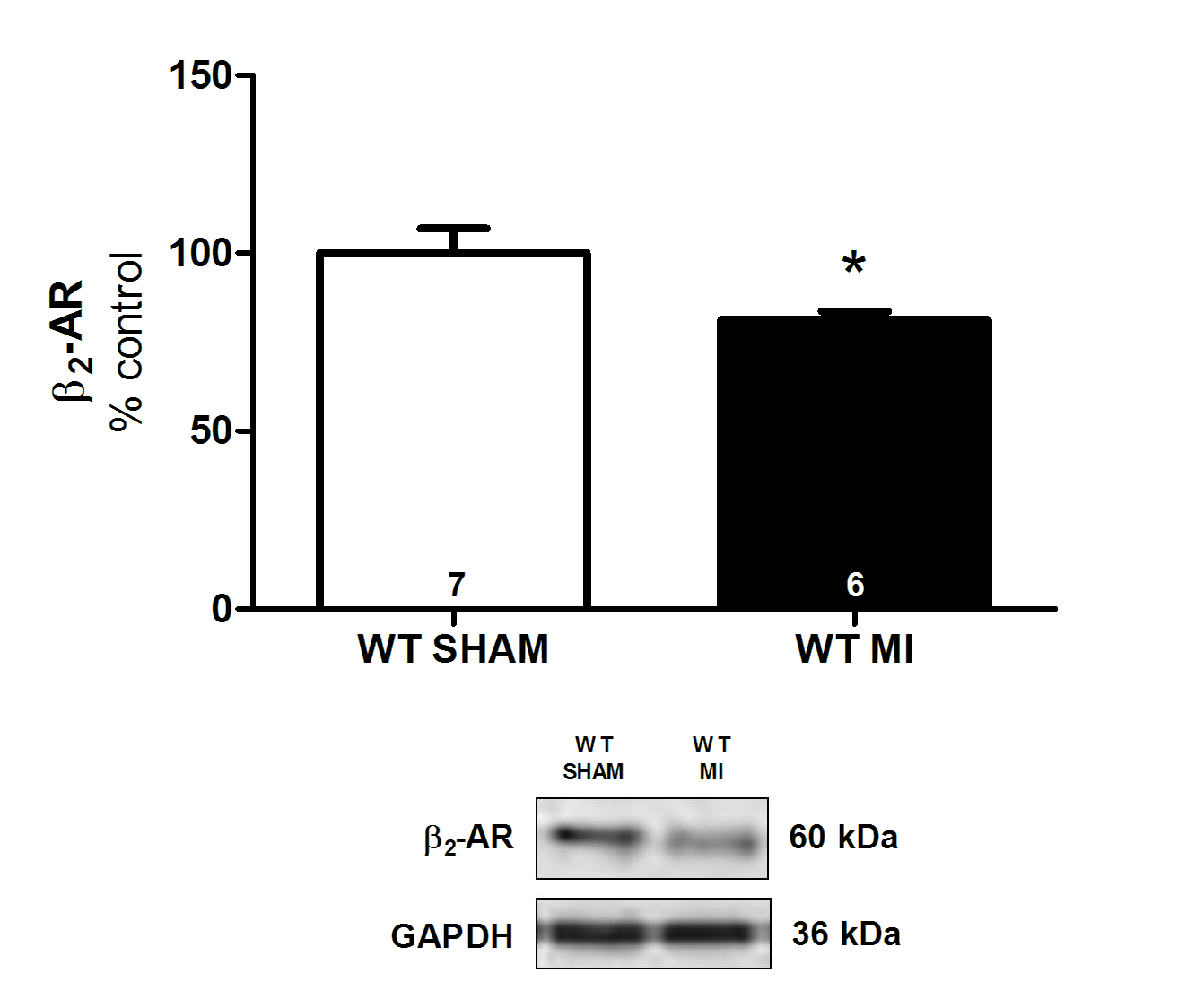

Supplement: Supplementary file 1 [file jcmm0018-1087-sd1.tif]

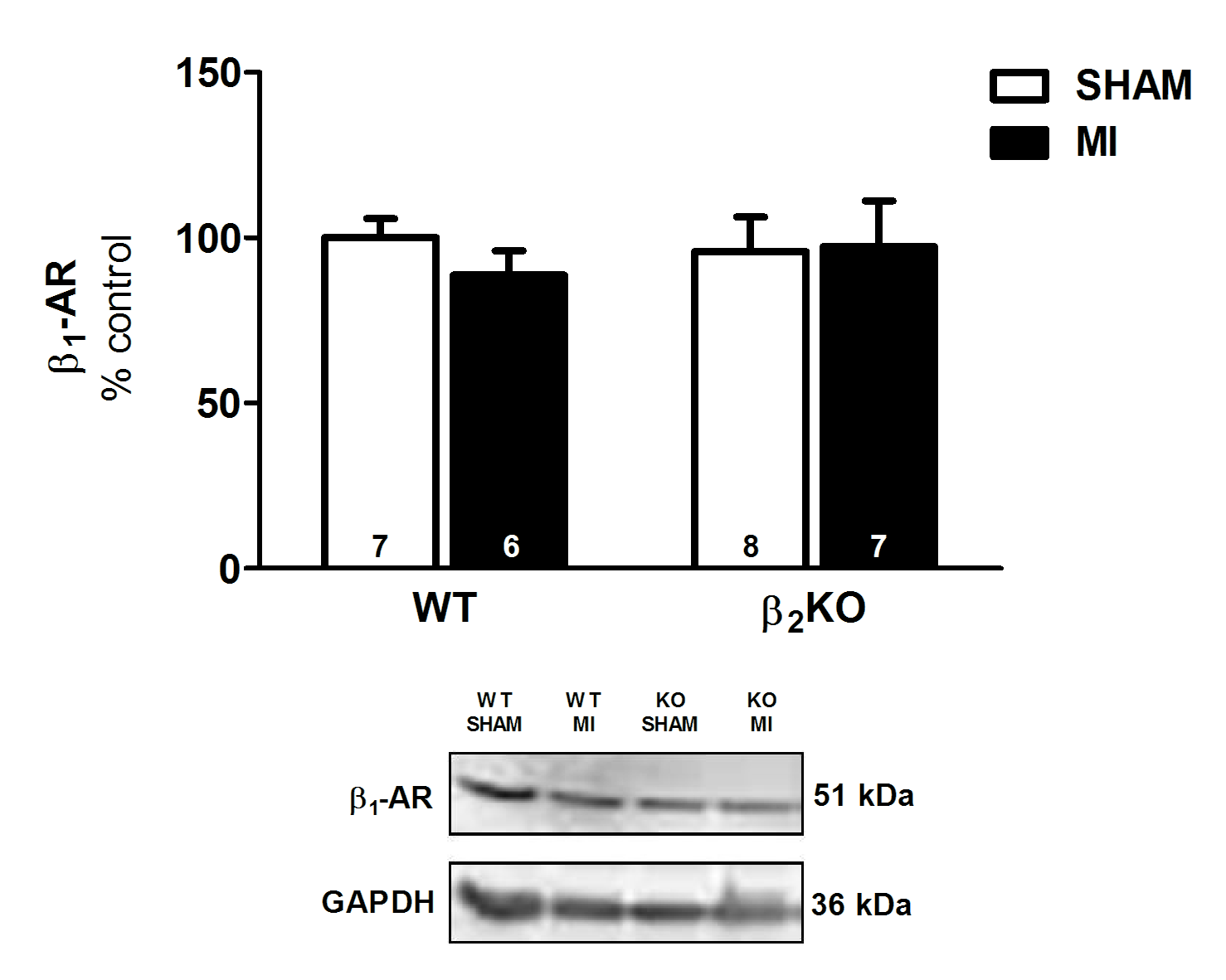

Supplement: Supplementary file 2 [file jcmm0018-1087-sd2.tif]
