# Supplementary material for: Lack of β2-adrenoceptors aggravates heart failure-induced skeletal muscle myopathy in mice
Source: J Cell Mol Med. 2014 Mar 13;18(6):1087–97. doi: 10.1111/jcmm.12253 (PMC4508148; doi:10.1111/jcmm.12253)
Supplement: Supplementary file 3 [file jcmm0018-1087-sd3.docx]

**ONLINE SUPPORTING INFORMATION FOR THE FOLLOWING JCMM ARTICLE:**

**TITLE:** Lack of β_2_-adrenoceptors aggravates heart failure-induced skeletal muscle myopathy in mice.

**AUTHORS:** Vanessa A. Voltarelli; Luiz R.G. Bechara; Aline V.N. Bacurau; Katt C. Mattos; Paulo M.M. Dourado; Carlos R. Bueno Jr.; Dulce E. Casarini; Carlos E. Negrao; Patricia C. Brum.

**Supplementary Information**

- **Supplementary Figures**


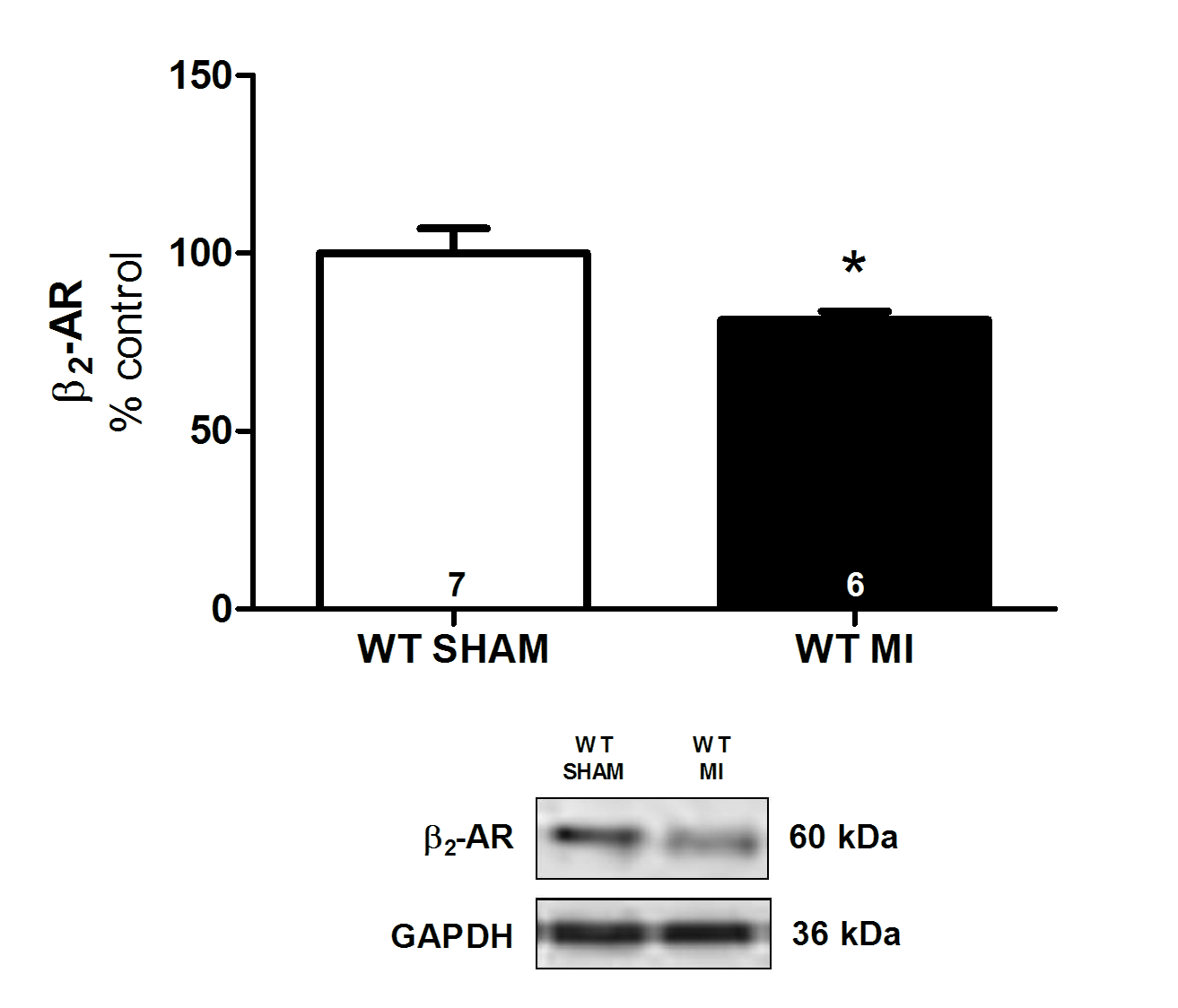


**Figure S1.** Plantaris β_2_-AR protein levels in WT mice submitted to SHAM or MI surgery. Data are presented as mean ± SEM. *p<0.05 *vs.* WT SHAM (Unpaired Student's *t*-test).


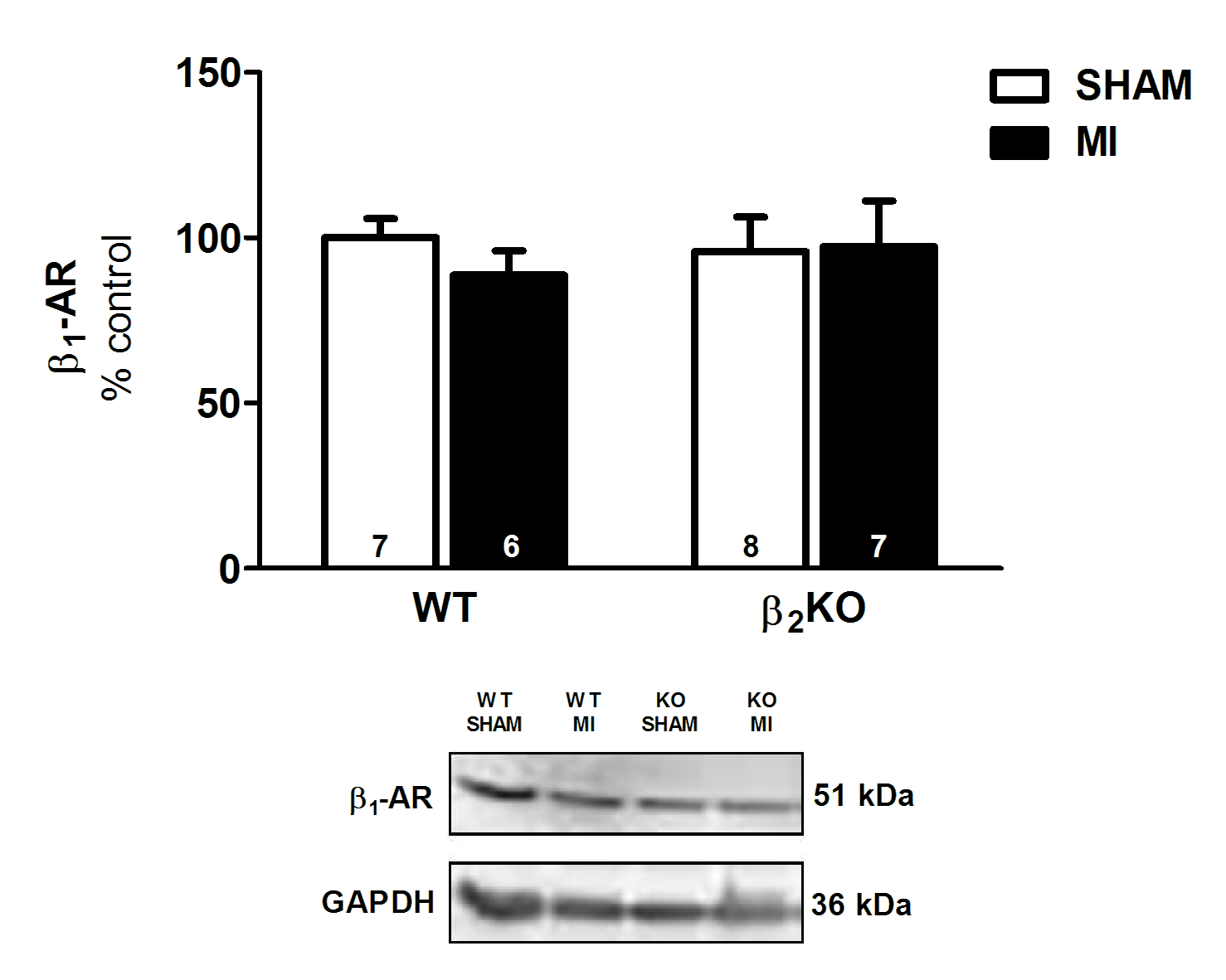


**Figure S2.** β_1_-AR protein levels in plantaris muscles of WT and β_2_KO mice from SHAM and MI groups. Data are presented as mean ± SEM.

- **Supplementary Methods**

**Plantaris protein expression of β_2_-AR and β_1_-AR**

Western blotting for β_2_-AR and β_1_-AR proteins were performed as described in the Methods. The following antibodies were used: Anti-beta 2 Adrenergic Receptor antibody (β_2_-AR, abcam®, Cambridge, UK) and Anti-beta 1 Adrenergic Receptor antibody (β_1_-AR, abcam®, Cambridge, UK). Binding of the primary antibody was detected with the use of peroxidase-conjugated secondary antibodies (anti-rabbit IgG e anti-mouse IgG, Cell Signaling Technology, Beverly, MA, USA, for 1.5 h at room temperature) and detection was performed in a digitalizing unit (ChemiDoc, BioRad, Hercules, CA, USA) after incubation with luminol and hydrogen peroxide as HRP substrate.
